# Identification and analysis of the germin-like gene family in soybean

**DOI:** 10.1186/1471-2164-11-620

**Published:** 2010-11-08

**Authors:** Mo Lu, Ying-Peng Han, Ji-Guo Gao, Xiang-Jing Wang, Wen-Bin Li

**Affiliations:** 1 Soybean Research Institute (Key Laboratory of Soybean Biology in Chinese Ministry of Education), Northeast Agricultural University, Harbin, PR China 150030; 2 Department of Life Science, Northeast Agricultural University, Harbin, PR China 150030

## Abstract

**Background:**

Germin and germin-like proteins constitute a ubiquitous family of plant proteins. A role of some family members in defense against pathogen attack had been proposed based on gene regulation studies and transgenic approaches. Soybean (*G. max *L. Merr.) germin genes had not been characterized at the molecular and functional levels.

**Results:**

In the present study, twenty-one germin gene members in soybean cultivar 'Maple Arrow' (partial resistance to *Sclerotinia *stem rot of soybean) were identified by in silico identification and RACE method (GmGER 1 to GmGER 21). A genome-wide analyses of these germin-like protein genes using a bioinformatics approach showed that the genes located on chromosomes 8, 1, 15, 20, 16, 19, 7, 3 and 10, on which more disease-resistant genes were located on. Sequence comparison revealed that the genes encoded three germin-like domains. The phylogenetic relationships and functional diversity of the germin gene family of soybean were analyzed among diverse genera. The expression of the GmGER genes treated with exogenous IAA suggested that GmGER genes might be regulated by auxin. Transgenic tobacco that expressed the GmGER 9 gene exhibited high tolerance to the salt stress. In addition, the GmGER mRNA increased transiently at darkness and peaked at a time that corresponded approximately to the critical night length. The mRNA did not accumulate significantly under the constant light condition, and did not change greatly under the SD and LD treatments.

**Conclusions:**

This study provides a complex overview of the GmGER genes in soybean. Phylogenetic analysis suggested that the germin and germin-like genes of the plant species that had been founded might be evolved by independent gene duplication events. The experiment indicated that germin genes exhibited diverse expression patterns during soybean development. The different time courses of the mRNAs accumulation of GmGER genes in soybean leaves appeared to have a regular photoperiodic reaction in darkness. Also the GmGER genes were proved to response to abiotic stress (such as auxin and salt), suggesting that these paralogous genes were likely involved in complex biological processes in soybean.

## Background

Germin is a protein marker that was first discovered in the germination of wheat seeds [1]. Subsequently, germin and germin-like proteins (GLPs) were found in other monocotyledonous, several dicotyledonous, angiosperms, gymnospermous plants, a myxomycete (slime mould) and *Physarum polycephalum *[2-10]. Germin relatives have also been identified in fern spores, prokaryotes and animals [11,12].

The germin family comprises a group of proteins belonging to a superfamily. All germins contain the germin motif that gives rise to a predicted β-barrel core involved in metal binding [13]. Most of them share biochemical attributes such as seed storage proteins, globulins and sucrose-binding, though they differ in their tissue specificities and enzyme activities [14-18]. The germin genes seemed to be involved in various important processes including development, osmotic regulation, photoperiodic oscillation, defence and apoptosis [19], and also founded to be associated with cell wall deposition [5,7,20,21].

Germin has an oxalate oxidase (EC 1.2.3.4) activity [1]. There has been growing evidence that germin encoded an enzyme that degraded oxalate to CO_2 _and H_2_O_2 _and also releases Ca^++ ^in some plant species. The degraded residual H_2_O_2 _plays different roles: a molecular signal for the induction of defence mechanisms, cross-linking of polymers in the extracellular matrix synthesis [9], and a direct antimicrobial effect, such as lignifications, to reinforce the cell walls [22-24]. The germin protein in monocotyledonous appeared to have an oxalate oxidase activity [21], but the germin-like proteins in dicotyledonous plants did not appear to have oxalate oxidase activity by 2010 [19]. For example, wheat and barley germin genes were found in the apoplast and the cytoplasm of germinating embryo cells with oxalate oxidase activity [21]. Two genes (gf-2.8 and gf-3.8) and a transcript (cDNA) of wheat germin have been sequenced [1].

Some germin genes may have functions other than oxalate oxidase activity [25]. Germin-like gene mRNAs have been found in leaves, cotyledons, stems, roots, embryos, flowers, seeds, and some were produced in response to environmental stimuli, depending on the species or the genes under consideration. Several evidences suggested that some GLPs have functions in general plant defence responses [26]. For instance, infection with pathogens, feeding of insects or application of chemicals such as salicylic acid, hydrogen peroxide (H_2_O_2_) or ethylene [27-32] could increase the expression of GLPs. In wheat and barley, transcription of at least one germin gene was induced upon a fungal infection [33]. Endogenous factors also controlled the expression of some germin genes since transcription of wheat germin gf-2.8 gene is stimulated by auxins [20]. Transient overexpression and transient silencing of certain barley GLP genes resulted in enhanced resistance to the powdery mildew fungus [17]. The promoter variant of rice oxalate oxidase genes played a role in resistance to *Magnaporthe oryzae *[34]. For some subfamilies, transient and stable expression showed a superoxide dismutase activity (EC1.15.1.1) of the encoded protein [31]. Silencing of a *Nicotiana attenuata *GLP increased the performance of an herbivore [30]. mRNA levels of mustard (*Brassica napus*) and a closely related Arabidopsis germin gene fluctuated during the circadian cycle [5,8].

*Sclerotinia *stem rot (SSR) caused by *Sclerotinia sclerotiorum *(Lib.) De Bary is a serious fungal disease of soybean. Since oxalic acid is a major pathogenic factor of SSR, transgenic soybean capable of degrading oxalic acid may be resistant to the pathogen [35]. To date, the wheat gf-2.8 gene has been studied on resistance to the oxalate-secreting pathogen *S. sclerotiorum*. Results showed transgenic soybean with the wheat germin gene greatly reduced disease progression and lesion length following cotyledon and stem inoculation with *S. sclerotiorum*, indicating that the germin gene products conferred resistant to *Sclerotinia *stem rot [35].

The GLPs with mostly unknown function in plant genomes [26] had been classified into subfamilies [36,37]. For example, the true germin subfamily, such as wheat and barley germins, included proteins with oxalate oxidase activity. In contrast, both GLP subfamilies 1 and 2 contained examples of proteins with superoxide dismutase (SOD) activity. Subfamily 3 included the phosphodiesterase (EC3.1) activity described above, and more subdivisions had been proposed recently [38]. A key feature of the GLP-related subfamilies, including the germins [37], was the conservation of a motif derived from that of the cupin superfamily [36]. In barley, five GLP subfamilies have been described and named HvGER1 to HvGER5. In *Physcomitrella *patens germin-like proteins, two novel clades have been found, named bryophyte-subfamilies 1 and 2 [38].

In contrast to the advanced knowledge of the structure, cell biology and expression features of barley and wheat germins and GLPs, less was known about soybean germin and germin-like genes by 2010. In the present work, nearly 50,000 sequenced and annotated ESTs of soybean were analyzed to find GmGER-like sequences by amino acid sequence similarity, and GmGER-like sequences were elongated by RACE. Their locations were determined and compared to disease resistance QTL, and both cluster and phylogenetic analyses of the germin-like proteins were performed to describe the variations in the soybean gene family. The abiotic factors (auxin-IAA, salt, light treatments) was tested on GmGER genes to evaluate the complex biological processes related to soybean development.

## Results

### Mining of germin-like EST sequences from soybean database and the determination of full-length cDNA sequences of GmGER genes

**Figure 1:**
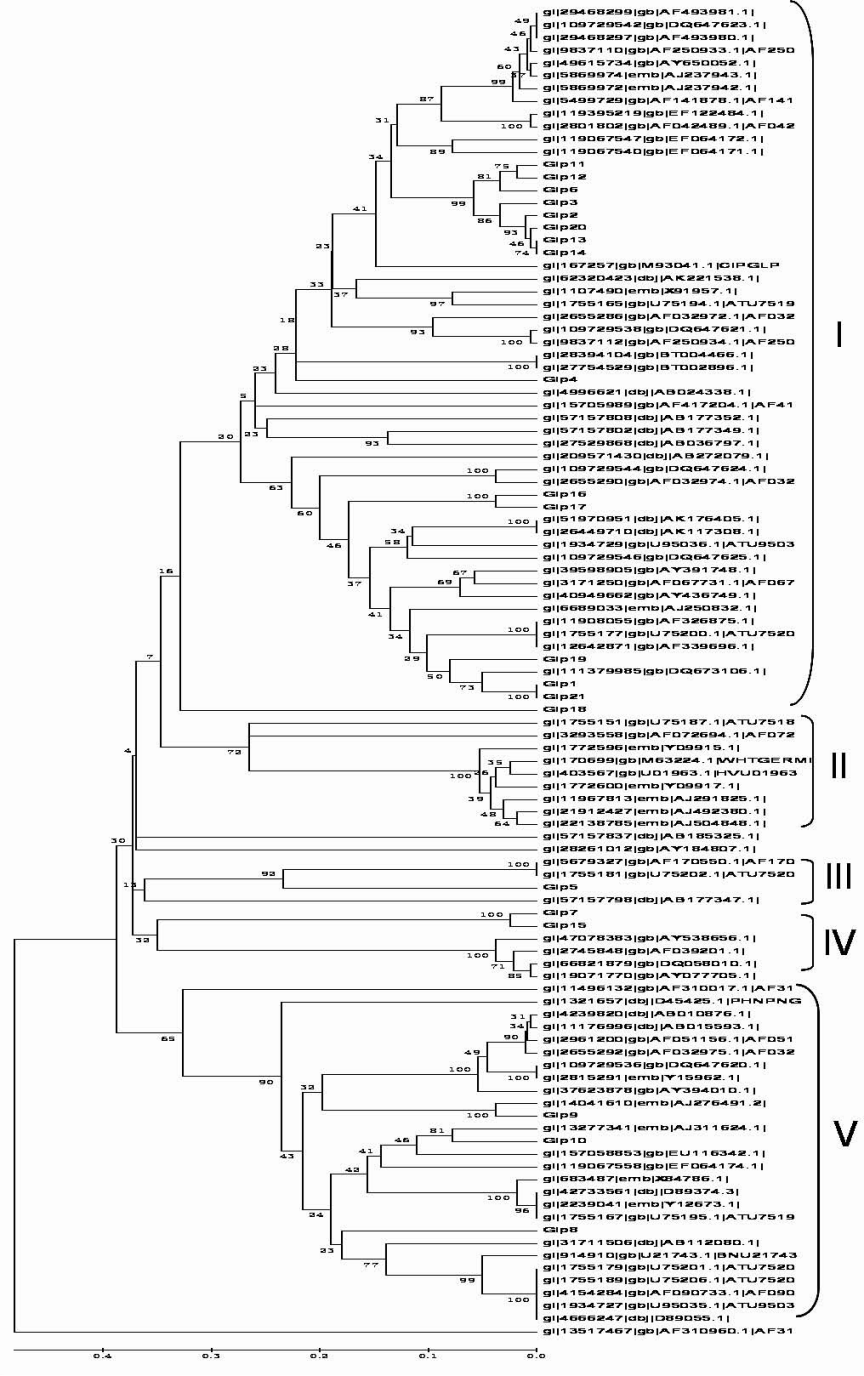
**Unrooted phylogenetic tree was constructed using the coding sequences of the GmGER genes and those of different plant species**. Bootstrap values were placed at the nodes and the scale bar corresponded to 0.1 estimated nucleic acid substitutions per site. Five major classes (I to V) were shown.

**Figure 2:**
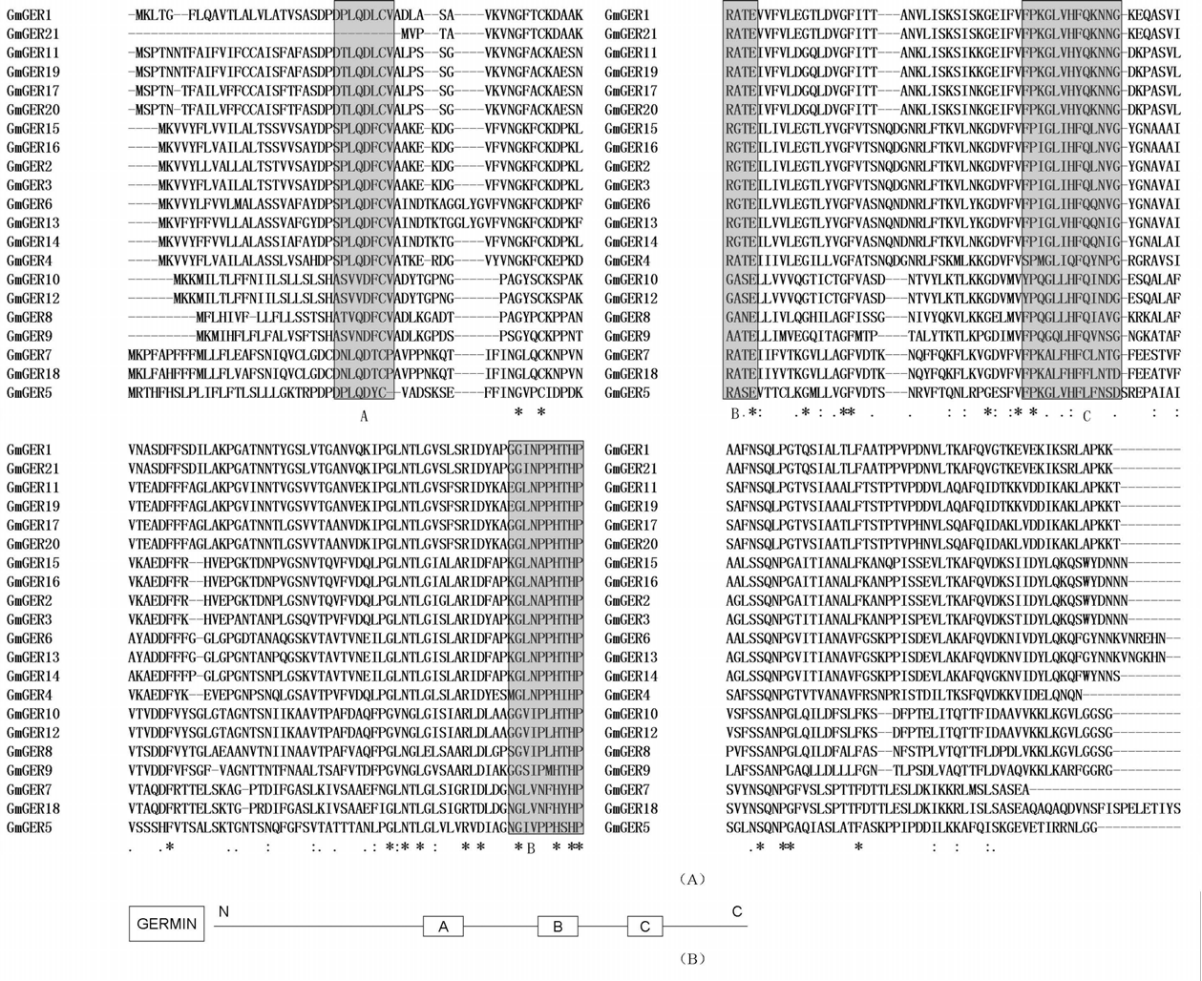
**The alignment of the germin domain in soybean germin-like genes and schematic diagram of soybean GmGER genes**. (A) The alignment of soybean germin-like protein domain was performed using the ClustalW program. (B) The rectangular boxes indicated the domains and their localization in each protein sequence.

123 soybean germin and germin-like EST sequences were detected by BLASTP and TBLASTN searches against the GenBank database. In addition, 204 nucleic acid sequences (both of the genes and ESTs) of germin and germin-like genes from 30 other plant species in the databases were collected for comparison to soybean data [Figure 1, Table 1 and 2]. After subsequent survey of soybean genomic data, twenty-one soybean germin-like ESTs or full length of cDNA genes were obtained. Of them two full-length mRNA was isolated by RACE. These genes were named as GmGER 1 to GmGER 21 and registered in NCBI GenBank [Table 2]. The open reading frames (ORFs) of the germin-like protein encoding genes were complementary DNA, and each gene had one exon. Most of the genes were transcribed from a single exon. The analyses of germin-like gene domain revealed that all of the GmGER genes had the similar domains that were lineated up closely each other [Figure 2] except the different position. The structure contained three boxes that represented the germin domains. The overall analyses revealed that the 21 proteins that contained the germin domains formed a single germin-like family in soybean.

**Table 1:** Accession numbers of germin family gene and protein sequences used in phylogenetic analyses

Gene Name	**Accession No.**^**a**^	Plant species	Gene Name	**Accession No.**^**a**^	Plant species
OsGLP1	AB010876 AB015593	*Oryza sativa*	GER1	EF064171	*Vitis vinifera*

KCS334B01	EF122484	*Oryza sativa*	GER2	DQ673106	*Vitis vinifera*

GER1	AF032971	*Oryza sativa*	GER3	AY298727	*Vitis vinifera*

GER2	AF032972	*Oryza sativa*	GER4	EF064172	*Vitis vinifera*

GER4	AF032974	*Oryza sativa*	GER5	EF064173	*Vitis vinifera*

GER5	AF032975	*Oryza sativa*	GER6	EF064174	*Vitis vinifera*

GER6	AF032976	*Oryza sativa*	GER7	EF064175	*Vitis vinifera*

GER7	AF072694	*Oryza sativa*	GLP	EU116342	*Chimonanthus praecox*

RGLP1	AF141880 AF141878	*Oryza sativa*	PnGLP	D45425	*Ipomoea nil*

RGLP2	AF141879	*Oryza sativa*	glp1	AY394010	*Zea*

GLP2a	U75192	*Oryza sativa*	GLP4	AY650052	*Triticum monococcum*

GLP3b	U75193 U75195	*Oryza sativa*	GLP	M21962	*Triticum aestivum*

GLP3b	U75193 U75195	*Oryza sativa*	Glp3	Y09917	*Triticum aestivum*

GLP16	AF042489	*Oryza sativa*	Glp1	Y09915	*Triticum aestivum*

GLP110	AF051156	*Oryza sativa*	Glp2b	AJ237943	*Triticum aestivum*

ger1	AJ250832	*Pisum sativum*	Glp2a	AJ237942	*Triticum aestivum*

glp3	AJ311624	*Pisum sativum*	GerA	AF250933	*Hordeum vulgare*

9f-3.8	M63224	Wheat	GerB	AF250934	*Hordeum vulgare*

9f-2.8	M63223	Wheat	GerD	AF250936	*Hordeum vulgare*

RmGLP1	AB272079	*Rhododendron mucronatum*	GerF	AF250935	*Hordeum vulgare*

RmGLP2	AB272080	*Rhododendron mucronatum*	GLP1	Y15962	*Hordeum vulgare*

Ger	AY436749	*Nicotiana attenuata*	GL8	AF493980	*Hordeum vulgare*

glp	AB112080	*Nicotiana tabacum*	GL12	AF493981	*Hordeum vulgare*

oxO1	AJ291825	*Lolium perenne*	GER1a	DQ647619	*Hordeum vulgare*

oxO2	AJ492380	*Lolium perenne*	GER2a	DQ647620	*Hordeum vulgare*

oxo3	AJ504848	*Lolium perenne*	GER3a	DQ647621	*Hordeum vulgare*

oxO4	AJ492381	*Lolium perenne*	GER4c	DQ647622	*Hordeum vulgare*

Glp1	X84786	*Sinapis alba*	GER4d	DQ647623	*Hordeum vulgare*

Glp	AY391748	*Capsicum annuum*	GER5a	DQ647624	*Hordeum vulgare*

Glp1	AY184807	*Medicago truncatula*	GER6a	DQ647625	*Hordeum vulgare*

OXAOXA	AF067731	*Solanum tuberosum*	HvGLP1	Y15962	*Hordeum vulgare*

BuGLP	AB036797	*Barbula unguiculata*	CM 72	U01963	*Hordeum vulgare*

Ger171	AF310017	*Musa acuminata*	GER1	DQ058010	*Larix x marschlinsii*

GLP	AB024338	*Atriplex lentiformis*	glp1	AJ276491	*Phaseolus vulgaris*

CIPGLP	M93041	*Mesembryanthemum crystallinum*	BNU21743	U21743	*Brassica napus*

Ger171	AF310017	*Beta vulgaris oxalate*	PcGER1	AF039201	*Pinus caribaea*

Ger165	AF310016	*Beta vulgaris oxalate*	GER1	AY077705	*Pinus sylvestris*

p2-A	AF310960	*Linum usitatissimum*	GER1	AY077704	*Pinus caribaea*

PpGLP1a PpGLP1b	AB177646 AB177347	*Physcomitrella patens subsp*	GLP1	AY538656	*Pinus taeda*

PpGLP2	AB185322 AB177348	*Physcomitrella patens subsp*	At1g09560	AF339696 AF326875	*Arabidopsis thaliana*

PpGLP3a	AB177349	*Physcomitrella patens subsp*	At3g04200	BT004466 BT002896	*Arabidopsis thaliana*

PpGLP3b	AB177645	*Physcomitrella patens subsp*	RAFL07	AK221538	*Arabidopsis thaliana*

PpGLP4	AB185323 AB177350	*Physcomitrella patens subsp*	RAFL24	AK176405	*Arabidopsis thaliana*

PpGLP5	AB185324 AB177351	*Physcomitrella patens subsp*	AtGER2	X91957	*Arabidopsis thaliana*

PpGLP6	AB185492 AB177352	*Physcomitrella patens subsp*	AtGLP1	D89055	*Arabidopsis thaliana*

PpGLP7	AB177353 AB185325	*Physcomitrella patens subsp*	AtGLP2	D89374	*Arabidopsis thaliana*

GLP1	NM_105920 F090733 U75190 U75189 U75196 U75197 U75201 U75206 U95034 U95035	*Arabidopsis thaliana*	GL22	NM_001083981	*Arabidopsis thaliana*

GLP3	NM_122070 Y12673	*Arabidopsis thaliana*	AT3G05950	NM_111469	*Arabidopsis thaliana*

GLP4	NM_101754 U75187	*Arabidopsis thaliana*	AT5G26700	NM_180544	*Arabidopsis thaliana*

GLP5	NM_100827 U75191 U75198 U75200	*Arabidopsis thaliana*	AT5G38910	NM_123253	*Arabidopsis thaliana*

GLP6	NM_123272	*Arabidopsis thaliana*	AT5G38930	NM_123255	*Arabidopsis thaliana*

GLP7	NM_100920 AF170550	*Arabidopsis thaliana*	AT5G38940	NM_123256	*Arabidopsis thaliana*

GLP8	NM_111467	*Arabidopsis thaliana*	AT5G38950	NM_123257	*Arabidopsis thaliana*

GLP9	NM_117545	*Arabidopsis thaliana*	AT5G38960	NM_123258	*Arabidopsis thaliana*

GLP10	NM_116067 NM_202748	*Arabidopsis thaliana*	AT5G39110	NM_123273	*Arabidopsis thaliana*

GLP2a	NM_001125862 NM_123281 U75192	*Arabidopsis thaliana*	AT5G39120	NM_123274	*Arabidopsis thaliana*

GLP3a	U75188 U75203	*Arabidopsis thaliana*	AT5G39130	NM_123275	*Arabidopsis thaliana*

GLP3b	U75193 U75195	*Arabidopsis thaliana*	AT5G39150	NM_123277	*Arabidopsis thaliana*

AT1G18980	NM_101755	*Arabidopsis thaliana*	AT5G39160	NM_001036906	*Arabidopsis thaliana*

AT3G10080	NM_111843	*Arabidopsis thaliana*	AT5G39180	NM_123280	*Arabidopsis thaliana*

AT3G04150	NM_111286	*Arabidopsis thaliana*	ATU75194	U75194	*Arabidopsis thaliana*

AT3G04170	NM_111288	*Arabidopsis thaliana*	ATU75202	U75202	*Arabidopsis thaliana*

AT3G04180	NM_111289	*Arabidopsis thaliana*	ATU75207	U75207	*Arabidopsis thaliana*

AT3G04190	NM_111290	*Arabidopsis thaliana*	ATU95036	U95036	*Arabidopsis thaliana*

AT3G04200	NM_111291	*Arabidopsis thaliana*	At1g02335	AK117308	*Arabidopsis thaliana*

^a ^Gene sequences were acquired from the NCBI sequence database http://www.ncbi.nlm.nih.gov/. Germin sequences from crop plants with published gene and/or protein expression data were included in the analysis.

**Table 2:** List of the amino acid lengths of the 21 GmGER genes and their numbers in NCBI

Gene name	Accession number (Genebank)	Protein ID (Genebank)	Amino acid length
GmGER 1	EU916269	ACL14493	1000
GmGER 2	EU916250	ACG69478	666
GmGER 3	EU916251	ACG69479	666
GmGER 4	EU916252	ACG69480	648
GmGER 5	EU916253	ACG69481	663
GmGER 6	EU916254	ACG69482	699
GmGER 7	EU916255	ACG69483	636
GmGER 8	EU916256	ACG69484	630
GmGER 9	EU916257	ACG69485	627
GmGER 10	EU916258	ACG69486	642
GmGER 11	EU916259	ACG69487	699
GmGER 12	EU916260	ACG69488	669
GmGER 13	EU916261	ACG69489	666
GmGER 14	EU916262	ACG69490	666
GmGER 15	EU916263	ACG69491	696
GmGER 16	EU916264	ACG69492	666
GmGER 17	EU916265	ACG69493	663
GmGER 18	EU916266	ACG69494	696
GmGER 19	EU916267	ACG69495	657
GmGER 20	EU916268	ACG69496	666
GmGER 21	EU925816	ACG69497	558

### Chromosomal locations of GmGER genes

**Figure 3:**
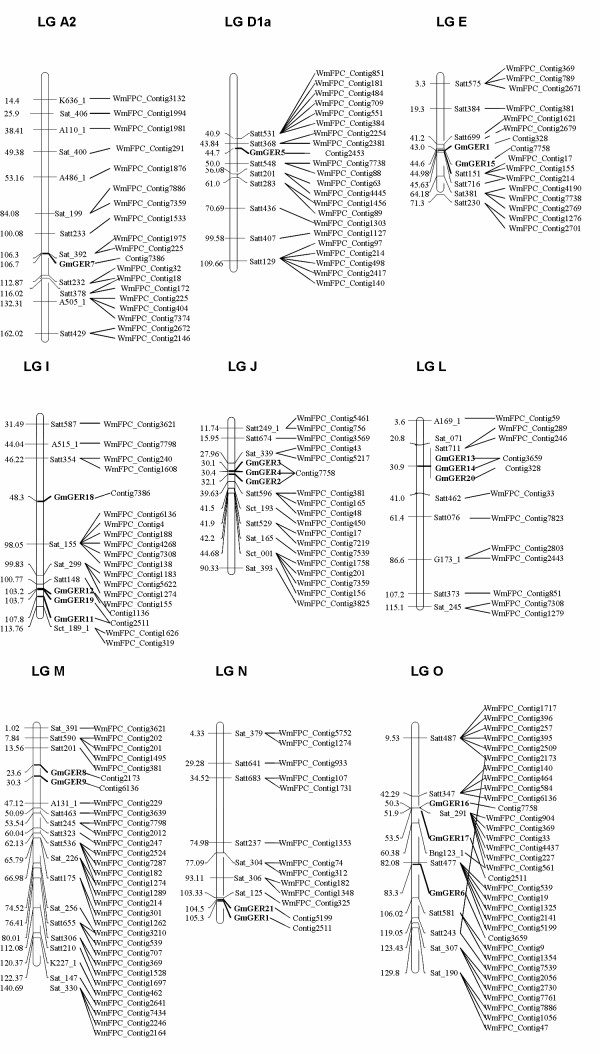
**Genomic arrangement and orientation of soybean germin-like protein genes on linkage groups (chromosomes) A2 (8), D1a (1), E (15), I (20), J (16), L (19), M (7), N (3) and O (10)**. Arrows indicated the transcription orientation of the genes. The numbers represented the exact positions of each gene. The QTL name and position referred to the Soybean Breeders Toolbox. The lines indicated the discrepancies of marker alignments between the physical map and genetic map.

Twenty-one genes of the soybean germin-like proteins were distributed on chromosomes (CH) 8, 1, 15, 20, 16, 19, 7, 3 and 10, respectively. An example of integration of the genetic and physical map with genetic markers for a detailed region of ~8 cM length on linkage group (LG) is illustrated in Figure 3. Among them, GmGER 7 was located at 106.7 cM on LG A2 (CH 8). GmGER 5 was located at 44.7 cM on LG D1a (CH 1). GmGER 10 and GmGER 15 were located at 43.0 and 44.6 cM on LG E (CH 15). GmGER 11, GmGER 12, GmGER 18 and GmGER 19 were located at 107.8, 103.2, 48.3 and 108.7 cM on LG I (CH 20), respectively. GmGER 2, GmGER 3 and GmGER 4 were located at 32.1, 30.1 and 30.4 cM on LG J (CH 16). GmGER 14 and GmGER 20 were located at 30.9 cM on LG L (CH 19). GmGER 6 and GmGER 9 were located at 23.6 and 30.3 cM on LG M (CH 7). GmGER 1 and GmGER 21 were located at 104.5 and 105.3 cM on LG N (CH 3). GmGER 6, GmGER 16 and GmGER 17 were located at 83.3, 50.3 and 53.5 cM on LG O (CH 10). There were several locations where single germin gene was dispersed and other genes appeared clustered. From the alignments between genetic markers and FPC contigs, few discrepancies of marker order between physical and genetic maps were obvious. The 21 genes distributed to 17 FPC contigs. Some of the genes in each of the clusters were transcribed from the same strand, indicating that they might have arisen by direct duplication [Figure 3]. However, each gene in the cluster had miner difference in the nucleic acid structure, which might happen during soybean evolution.

### Multiple sequence alignment and phylogenetic tree for soybean germin gene family

To uncover the common characteristics of proteins in germin-like gene families, the predicted protein sequences of the 21 soybean GmGER genes was compared [Figure 2]. All these proteins possessed one or two N-glycosylation consensus sequences. Three other conserved regions were found and might have important functions in these proteins. However, no precise function had been attributed to them to date. The three domains were located in the 3' end of the larger protein coding germin genes. The structure consensus sequence of motif A was HTHPRATEILTVLEGTLYVGF with 21 amino acids. The structure consensus sequence of motif B was KVLNKGDVFVFPEGLIHFQFN with 21 amino acids. The structure consensus sequence of motif C was [N/S]SQNPGIVFVPLTLFG with 16 amino acids.

**Figure 4:**
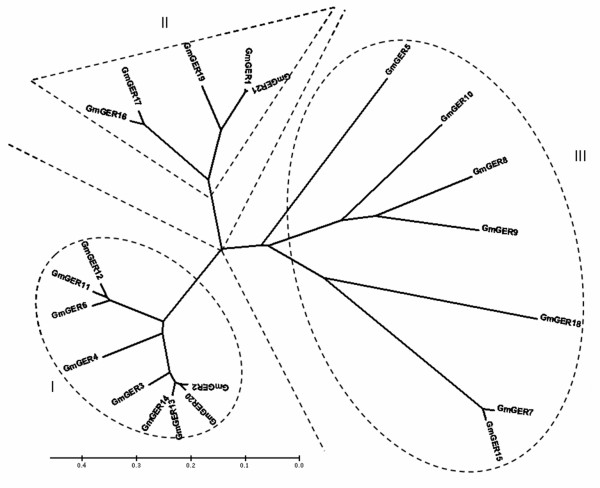
**Unrooted Bayesian tree of soybean GmGER genes**. Bootstrap values were placed at the nodes and the scale bar corresponded to 0.2 estimated nucleic acid substitutions per site. Three major classes (I, II and III) were shown.

A phylogenetic relationship of GmGER with the paralogous RNA helicases was inferred by constructing the neighbor-joining tree. The tree topology was found to be well aligned with the 21 evolutionary lineage among soybean derived from a broad array of clades. Also, the binary alignment analysis of the 21 GmGER genes with each predicted paralogous genes yielded consistent results supported by high sequence identity values. A RNA helicase protein, GmGER 2 and GmGER 20 exhibited the highest sequence identity with GmGER 13 and GmGER 14. GmGER 6 and GmGER 11 exhibited the highest sequence identity with GmGER 12. GmGER 1 exhibited the highest sequence identity with GmGER 21. GmGER 16 exhibited the highest sequence identity with GmGER 17. GmGER 8 exhibited the highest sequence identity with GmGER 9. GmGER 7 exhibited the highest sequence identity with GmGER 15 [Figure 4]. Phylogenetic analyses showed that all the GmGER genes were relatively tightly clustered together. Notably, these sequences were most closely related with those proteins sharing the same domains. The genes in the same group were closed to each other and far off the genes from the other groups. Therefore, the genes in the three groups might originate from different ancestors.

### Comparison of soybean germin family with other species by phylogenetic tree

To study the phylogeny of the GmGER gene family, the complete germin protein sequences of different species were collected. Redundant or highly similar sequences were removed and a phylogenetic tree with 105 complete GLP sequences was constructed with the neighbor-joining method [Figure 1]. The results showed a complex evolutionary history existed with recent gene duplications of soybean due to the difficulty to identify orthologs of particular soybean germins in other species. However, subfamilies of germin genes had been described. A few clades [5] representing different species emerged. The germin-like gene family proteins in soybean and in other species were apparently divided into different clades [Figure 1]. The relationship among gene lineages were roughly congruent with established phylogenetic relationship among taxa. For example, relationships among gene lineages in the clade I subfamily corresponded closely with phylogenetic relationships among Arabidopsis, rice, *Pisum sativum*, *Vitis vinifera*, *Hordeum vulgare*, *Triticum aestivum*, *Atriplex lentiformis*, *Musa acuminate*, *Rhododendron mucronatum*, barley and wheat. The results from phylogenetic tree suggested that the conservation of tissue specific expression pattern existed within a few subfamilies.

The GmGER genes fell into five major clades. Clade I contained the largest number of the GLP subfamilies, including 15 members of the 21 GmGER genes. GmGER 1 was closely related to *Vitis vinifera *and *Pisum sativum*. GmGER 2 was closely related to *Hordeum vulgare*. GmGER 3 was closely related to *Hordeum vulgare*. GmGER 4 was closely related to *Atriplex lentiformis *and *Musa acuminate*. GmGER 6, GmGER 11, GmGER 12, GmGER 13, GmGER 14 and GmGER 20 were closely related to *Triticum aestivum*. GmGER 16 and GmGER 17 were closely related to *Rhododendron mucronatum*. GmGER 19 was closely related to *Pisum sativum*. GmGER 21 was closely related to *Pisum sativum *and *Vitis vinifera*.

Clade II did not contain any GmGER gene. Included in this clade were Arabiopsis GLP gene members, two rice GLP genes, four barley GLP genes, one wheat GLP gene and three *Lolium perenne *GLP genes. This suggested that clade II was conserved in grasses. Clade III contained four members, including GmGER 5 gene, two Arabiopsis GLP genes and one *Physcomitrella *patens subsp. patens GLP gene. Clade III was conserved in both dicot and monocot plants. Clade IV contained six members, GmGER 7, GmGER 15, three Pinus GLP genes and one Larix x marschlinsii (hybrid larch) GLP gene. It seemed that GmGER 7 and GmGER 15 were closely related to Pinus GLP genes. GmGER 8, GmGER 9 and GmGER 10 were covered in clade V, in that GLPs were widely dispersed in different plant species. GmGER 9 and *Phaseolus vulgaris *GLP genes were homologously grouped. GmGER 10 and *Pisum sativum *GLP gene were closely related to Chimonanthus praecox GLP gene and *Vitis vinifera *GLP gene. The three GmGER genes were also closely related to *Beta vulgaris *GLP gene and *Linum usitatissimum *cv. GLP genes.

### mRNA expression of GmGER genes in response to auxin-IAA

**Figure 5:**
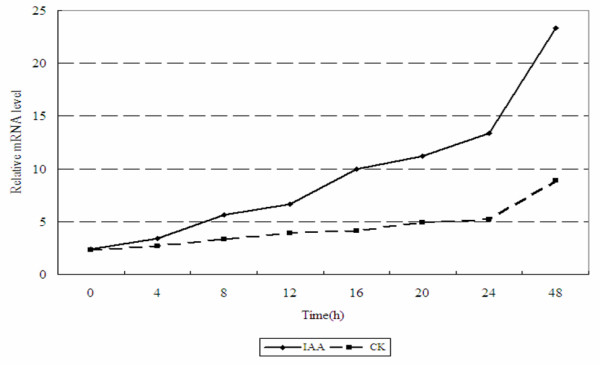
**Expression analysis of the GmGER gene by quantitative real-time RT-PCR**. The analyse is in response to IAA (100 u M) treatment at 0, 4, 8, 12, 16, 20, 24, 48 hours.

The expression of the GmGER genes was dramatically stimulated within 8 h after the addition of IAA, maintained for 12 h, and then decreased gradually, according to the results of real-time RT-PCR [Figure 5], suggesting that GmGER genes might be regulated by auxin.

### mRNA expression of GmGER genes under light stress

**Figure 6:**
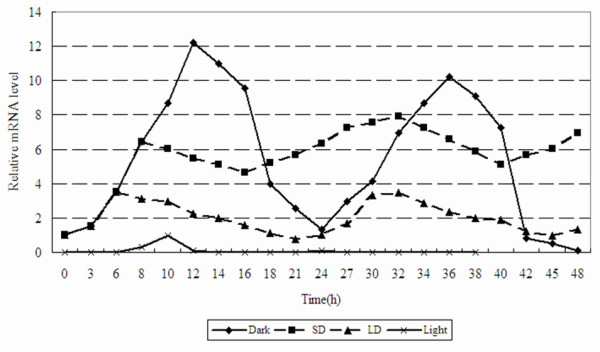
**Expression level of GmGER mRNA in four photoperiodic treatments**. Continuous darkness (up to 48 h), continuous light (up to 48 h), SD (short-day, 8 h light and 16 h dark), LD (long-day, 16 h light and 8 h dark).

Changes in the expression level of GmGER mRNA in leaves, under darkness, long day (LD) and short day (SD) treatments, were examined by quantitative real-time RT-PCR. The level of GmGER mRNA showed an obvious increase and a decrease during 48 h of continuous darkness (peaked at 12 h and 36 h), suggesting the existence of a circadian clock feature [Figure 6]. The expression of GmGER mRNA was not clear during the continuous light for 48 h. The level of GmGER mRNA showed a moderate changes in SD and LD treatments (SD treatment being higher than LD treatment) [Figure 6].

### Salt tolerance of transgenic tobacco with GmGER 9 gene

**Figure 7:**
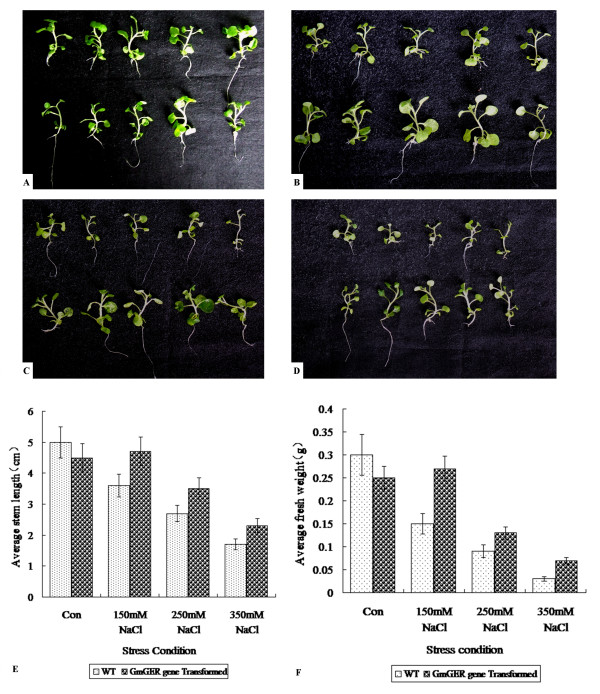
**Seedlings of transgenic tobacco with GmGER gene and WT tobacco under different concentration of NaCl stress**. A: normal growth condition; B: 150 mM NaCl stress; C: 250 mM NaCl stress; D: 350 mM NaCl stress; E: comparison of average stem length between transgenic and WT plants; F: comparison of average fresh weight between transgenic and WT plants. The first lines in the A, B, C, D were WT tobacco; the second lines in the A, B, C, D were the tobacco plants transformed with GmGER gene.

Both fresh weight and stem length of transgenic tobacco plants were significantly higher than in the WT plants after exposure to 150, 250 and 350 mM of NaCl [Figure 7B, 7C, 7D, 7E and 7F]. Under the treatment of 350 mM NaCl, the WT plants grow smaller and yellower, whereas, the GmGER 9 transformed plants grow taller [Figure 7A, 7B, 7C and 7D]. There were no differences in fresh weight and stem length between WT and transgenic plants without NaCl supplement [Figure 7A, 7E and 7F].

## Discussion

Germin genes form a large family and their functions are still under studying. In this study, we identified 21 germin genes of soybean. The analyses of gene domain revealed that the unknown GLP gene family and their functions might be determined according to the presence of germin domain. Interestingly, the predicted model almost perfectly conformed to the determined structure of individual domain of the seed storage globulins, canavalin and phaseolin that showed a significant sequence similarity with germins [39,40]. These proteins contain two or three similar domains that might have evolved following gene duplication events from a common ancestral gene or domain [40,41].

To date, no germin gene was identified in soybean. Thus, the 21 GmGER genes found in the present work were thought as the novel, species-specific proteins of soybean. Comparative analyses revealed that these evolutionary related germin genes shared very similar exon structures, suggested the close phylogenetic relationships among the soybean germin genes.

The common structural features of germin-like proteins included: conserved structural elements, secretory transit peptides, protein glycosylation sites and regions of conserved sequence similarity [36]. According to the review of Carter and Thornburg [36], the most important conserved structural feature among the GLPs is a conserved amino acid sequence termed the germin box. The consensus pattern is: GxxxxHxHPxAxEh, where x is any amino acid and h is a hydrophobic amino acid. Mature germins and GLPs all contain approximately 200 amino acids in length and the germin box occurs near the middle for all proteins in this family. In soybean, the conserved region B was found in all the 21 germin genes. Conserved region A enriched in hydrophobic amino acids always followed this peptide (GxxxxHxHPxAxEh) [Figure 2]. Another conserved region A was localized in the amino terminal part of the mature proteins, and strongly conserved in all the germins but not in the spherulins. It is likely that the heptapeptide sequence [L/V]QDFCV[A/G], found in all germin-like proteins examined, was of importance in the biochemical functions of these proteins. A third putative conserved region C underlined in Figure 2 was [V/M][F/K]P[Q/K/I]G[L][V/I/L]HFQ[K/L/Q/I]N[V/N/I]G. Two His residues and a Glu residue in Motif A, together with a His residue in Motif C, act as ligands for the binding of a manganese ion at the active site of the archetypal germin, cupin [42]. In our result, motif A was HTHPRATEILTVLEGTLYVGF with 21 amino acids, motif B was KVLNKGDVFVFPEGLIHFQFN with 21 amino acids, and motif C was [N/S]SQNPGIVFVPLTLFG with 16 amino acids. The 21 GmGER genes all have the germin activity, **e**xhibiting diverse expression patterns during soybean development, a regular photoperiodical reaction in darkness, response to abiotic stress (such as auxin and salt), as we have proved in this paper.

One question that remained to be answered was whether all germin or germin-like genes of soybean carried out the same functions and were regulated in the same way like in other species. Multi-sequence alignments of GLPs in barley and Arabidopsis, as well in other plant species showed some overlaps of multigene family structure, which would be helpful in functional annotation and the study of the evolutionary relationships among the genes [31]. Based on phylogenetic analysis, we concluded that the GmGER genes did not share a common expression pattern. Germin genes were functionally diverse but structurally related [43]. Large differences existed among several members of germin gene family. It was clear that soybean GmGER genes were homologous to the dicotyledon proteins in the phylogenetic tree (Figure 1). Sequence analyses revealed that GmGER 8, 9 and 10 were distinct from the other GmGER genes. The GmGER 8 and 9 were located on the same chromosome 7, suggesting that they might be divergence from a common ancestor. Likewise, the GmGER 2, 3, 6, 11, 12, 13, 14 and 20 were closely related to each other. It was possible that they were recently descended from a common ancestor gene that evolved into different forms.

Germin and the GLP gene family could be divided into two distinct groups. The members in one group (germins) had relatively homogeneous sequences [44], meanwhile, the members in another group (GLPs) were much more numerous and showed high sequence divergence [45]. GLPs could be further divided into three subgroups based upon sequence conservation [37]. In this paper, the GmGER genes were also divided into three subgroups based upon sequence conservation. Systematic investigation of soybean germin gene family would be useful for defining origins of the germin and germin-like gene family. Although the functional significance of all these elements remained to be tested, a variety of regulatory mechanisms acting on the transcript abundance were found. The germin genes in rice have been confirmed to be expressed in all types of tissues and could be induced by biotic or abiotic stresses. Many of the stress-induced germin genes were physically co-localized with quantitative trait loci (QTL) for disease resistance, and the emerged evidence suggested that microRNAs might regulate their transcript abundances [46,47]. Oxalate oxidase could confer to enhance resistance to *Sclerotinia *blight in peanut [41]. In transformed sunflower, the expression of oxalate oxidase resulted in the induction of plant defense proteins [48]. In tissue sections derived from pea nodules, PsGER1 was shown to be the first known germin-like protein with superoxide dismutase activity [49].

A significant promotion of the expression of GmGER mRNA was achieved by quantitative real-time RT-PCR under the supplement of IAA in medium. As IAA is involved mainly in the regulation of cell elongation and stimulating cell division [50], this result suggested that GmGER genes might mediate the stimulating effect of auxin on cell division. It should be noticed that, although the GmGER gene responded to IAA, the affinity was unknown, which made it difficult to be certain that the GmGER gene was involved in auxin signaling, especially without additional physiologic evidence. Therefore, it should be investigated furthermore. Meanwhile, the assay of salt stress indicated that the transgenic seedlings of tobacco with GmGER 9 gene showed improved salt tolerance compared to the WT plants (Figure 7A, 7B, 7C, 7D, 7E and 7F), confirming that the GmGER 9 gene had a positive response to the salt condition. Salt stress could cause oxidative damage in plant, such as protein oxidation and lipid peroxidation [51]. Therefore, GmGER 9 gene remained a potential to improve the salt tolerance in transgenic plants.

Different families of genes have been reported to be associated with plant photoperiod including germin genes [19]. A significant difference was observed in the response to light/dark cycles by various genes and different species. The level of mRNA of *Sinapis alba *L. undergoes circadian oscillation during light/dark cycles with a maximum about 12 h after the light was turned on. This peak of mRNA accumulation occurred in light treatment [52]. By contrast, the level of GmGER mRNA reached its maximum about 12 h after light was turned off in this study. The peak of mRNA accumulation occurred during the dark period in darkness treatment and changed sharply. In the light treatment the mRNA had almost no expression. In the LD treatment, the mRNA level had mild change less than in SD treatment. The value of mRNA was a little higher in SD treatment than in LD treatment. These findings indicated that the transcription of the GmGER genes greatly depended on the duration of darkness.

## Conclutions

In summary, 21 germin-like protein genes of soybean were identified and analyzed in this study. The results revealed that this novel family of germin-like proteins might represent an important mechanism in soybean to modulate diverse physiological and molecular processes. These findings provided the groundwork to assist functional studies of this novel GmGER family, and an opportunity to discover the roles of the germin family proteins, to underlie regulatory mechanisms during plant development and the responses to adverse environmental stimuli and to answer why several different genes were required to carry out these functions. Future studies using molecular, genetic, biochemical, physiological, and other approaches could provide insights into understanding the functions and elucidating the molecular mechanisms of soybean germin genes in plant defense responses and development.

## Methods

### Sequence data and database search

The nucleic acid sequences and EST sequences of germin and germin-like genes in *Glycine *max (L.) Merr. and in other species were searched from the GenBank database http://www.ncbi.nlm.nih.gov/Entrez/ with BLASTP and TBLASTN program in NCBI with e-value 10 [53]. Then all the sequence data of germin and germin-like genes were downloaded (EST sequences for searching soybean cDNA and other nucleic acid sequences for analyzing the relationship among soybean germin genes). In an attempt to obtain all of the germin and germin-like genes in soybean, the EST sequences were used to search the soybean genomic DNA databases, Phytozome http://www.phytozome.net/soybean.php. The predicted protein sequences of putative soybean germin family members were also downloaded from these databases. The software Genescan web server http://genes.mit.edu/GENSCAN.html was used for gene prediction. Redundant hits were removed by manual inspection. The InterProScan program http://www.ebi.ac.uk/InterProScan/ was used to detect the germin and germin-like domains.

### Full-length cDNA sequences and chromosomal location of germin genes

Here, a total of 123 soybean germin-like gene EST sequences were downloaded from the GenBank database. The coding regions of soybean candidates were used to perform a BLASTN search against all of the ESTs. If the hits showed a complete identity over the entire polypeptide, it was considered an entire and active gene in soybean. All the sequences that had been determined were used as query in BALSTN searches against the soybean genome data http://www.phytozome.net/soybean.php. The sequences of the 20 soybean linkage groups http://soybeanphysicalmap.org/[54] were also used as query in BALSTN searches against the soybean genome data http://www.phytozome.net/soybean.php. The two alignment results were used to calculate with Blastm.pl (edited by our laboratory), then arranged in Microsoft Office Excel 2003. The chart from these data was drawn by Mapchart http://www.kyazma.nl/index.php/mc.JoinMap/. The genes were placed by the position on the genetic map and the physical map.

The 5'- and 3'-rapid amplification of cDNA ends (RACE) was performed to obtain the 5'- and 3' ends encoding the additional sequence of soybean germin genes. The SMART RACE cDNA Amplification Kit (Clontech) was used following the manufacturer's instructions. Samples of 1 μg of total RNA from the leaves of soybean 'Maple arrow' were used for reverse transcription. The gene-specific primer GSP1 from the antisense strand was designed for 5'-RACE, and the gene-specific primer GSP2 from the sense strand was used for 3'-RACE. All RACE PCR reactions were performed using the following protocol: 94°C for 30 s, 70°C for 30 s and 72°C for 3 min for 5 cycles, followed by 94°C for 30 s, 68°C for 30 s and 72°C for 3 min for 5 cycles, followed by 94°C for 30 s, 66°C for 30 s and 72°C for 3 min for 27 cycles. The PCR product was subcloned into pGEM-T vector and sequenced.

### Multiple sequence alignment and phylogenetic tree construction

Multiple alignments of nucleic acid sequences were performed using ClustalW ([55]; http://www.ebi.ac.uk/clustalw/index.html) with the following parameters: gap opening penalty 10, gap extension penalty 1.0. The PAM series was used for the protein weight matrix. The results were represented with the help of the GeneDoc software [56]. Phylogenetic trees were constructed by the Neighbor Joining method using the MEGA3.0 program [57]. Each analysis was carried out at least twice.

### GmGER gene response to IAA stress

In order to evaluate GmGER gene response to IAA in soybean development, 'Maple arrow' seedlings were surface sterilized and then placed on the soil under aseptic condition, and kept in growth chamber under white fluorescent light (600 umol m^-2^s^-1^, 16 h light/8 h dark) at 25°C and 90% relative humidity. The seedlings without any treatment were used as the control. After germination for 7 days, the seedlings were transferred to plates supplemented with dosed IAA (100 uM) and grown for another 6 days. The seedlings were then sampled and frozen for further analysis.

### GmGER gene response to light/dark

Seeds of soybean 'Maple arrow' were surface sterilized and then placed on the soil under aseptic condition and then kept in growth chamber under white fluorescent light (600 umol m^-2^s^-1^, 16 h light/8 h dark) at 25°C and 90% relative humidity. When the cotyledons had opened maximally, the seedlings were subjected to one of the four photoperiodic treatments: dark (continuous darkness up to 48 h), light (continuous light up to 48 h), SD (short-day, 8 h light and 16 h dark), LD (long-day, 16 h light and 8 h dark).

### Salt tolerance assay

Seeds of transgenic tobacco with GmGER 9 gene were surface sterilized and then placed on MS medium [58] under aseptic condition and then kept in growth chamber under white fluorescent light (600 umol m^-2^s^-1^, 16 h light/8 h dark) at 25°C and 90% relative humidity. In the same time, the WT tobacco seeds were also planted on MS medium as a control. After germination, seedlings of transgenic plants and WT plants with similar size were transferred to MS medium containing different concentrations of NaCl (0, 150, 250 and 350 mM). After 30 d, the fresh weights of 5 to 10 seedlings from each transgenic line and WT plants in each treatment were measured, and the stem lengths of tested seedlings were also measured and compared.

### Quantitative real-time RT-PCR

Total RNA was isolated from the soybean leaves at 0, 4, 8, 12, 16, 20, 24, 48 hours after IAA treatment. The amplification of selected genes was performed by quantitative real-time RT-PCR with specific oligonucleotide primers: forward primer (TTCCTCTTTGCTCTTGTC) and reverse primer (AGTGTTTGTGGTGTTTCC), using the first strand cDNA. DNase treatment was given for removing contaminating genomic DNA from RNA samples. The PCR reactions (1× PCR buffer, 200 μm dNTPs, 150 ng of each gene specific primer, 5U Taq Polymerase and 1× SYBR-GreenR using Icycler (BioRad, USA) were carried out at 94°C for 1 min, 55°C for 1 min and 72°C for 1 min for 35 cycles. At the end of the PCR cycles, the products were analyzed through a melt curve analysis to check the specificity of the PCR amplification. Two replicates of each reaction were performed, and data were analyzed by Livak method [59] and expressed as normalized expression ratio (2^-ΔΔCT^) of particular gene to specific stress treatment. Expression ratio was calculated as ΔΔC_T _= ΔC_T _(gene) -ΔC_T _(β-tubulin); ΔC_T _(gene) = ΔC_T _(transgenic line) - ΔC_T _(CK plant); ΔC_T _(β-tubulin) = ΔC_T _(transgenic line) -ΔC_T _(CK plant).

## Authors' contributions

ML performed the preliminary molecular experiments, interpreted the results and wrote the manuscript. YPH and JGG participated in the design. XJW conceived the study. WBL revised the manuscript. All authors read and approved the final manuscript.
