# Identification and analysis of the germin-like gene family in soybean

**DOI:** 10.1186/1471-2164-12-16

**Published:** 2011-01-10

**Authors:** Mo Lu, Ying-Peng Han, Ji-Guo Gao, Xiang-Jing Wang, Wen-Bin Li

**Affiliations:** 1 Soybean Research Institute (Key Laboratory of Soybean Biology in Chinese Ministry of Education), Northeast Agricultural University, Harbin, 150030, PR China; 2 Department of Life Science, Northeast Agricultural University, Harbin, 150030, PR China

## Abstract

In line 12 of page 1, replace "GmGER 9" with "GmGER 15".

## Correction

After the publication of this work [1], we became aware of the fact that the gene name 'GmGER 9' was mistakenly used throughout the gene function analyses in the original manuscript due to an error between the gene identification number recorded in our notebook and the nearest matching GenBank accession. The gene name 'GmGER 9' should be changed to 'GmGER 15' in the following places:

## Results

In the last Paragraph of the results section (page 9), replace "GmGER 9" with "GmGER 15".

## Discussion

In line 2 of page 12, replace "GmGER 9" with "GmGER 15".

In line 5 of page 12, replace "GmGER 9" with "GmGER 15".

In line 8 of page 12, replace "GmGER 9" with "GmGER 15".

## Methods

In line 1 of the Paragraph "Salt tolerance assay" on page 13, replace "GmGER 9"

with "GmGER 15".
